# Supplementary figures and images for: Non-Invasive Pulsatile Shear Stress Modifies Endothelial Activation; A Narrative Review
Source: Biomedicines. 2022 Nov 28;10(12):3050. doi: 10.3390/biomedicines10123050 (PMC9775985; doi:10.3390/biomedicines10123050)

**Figure 1S**

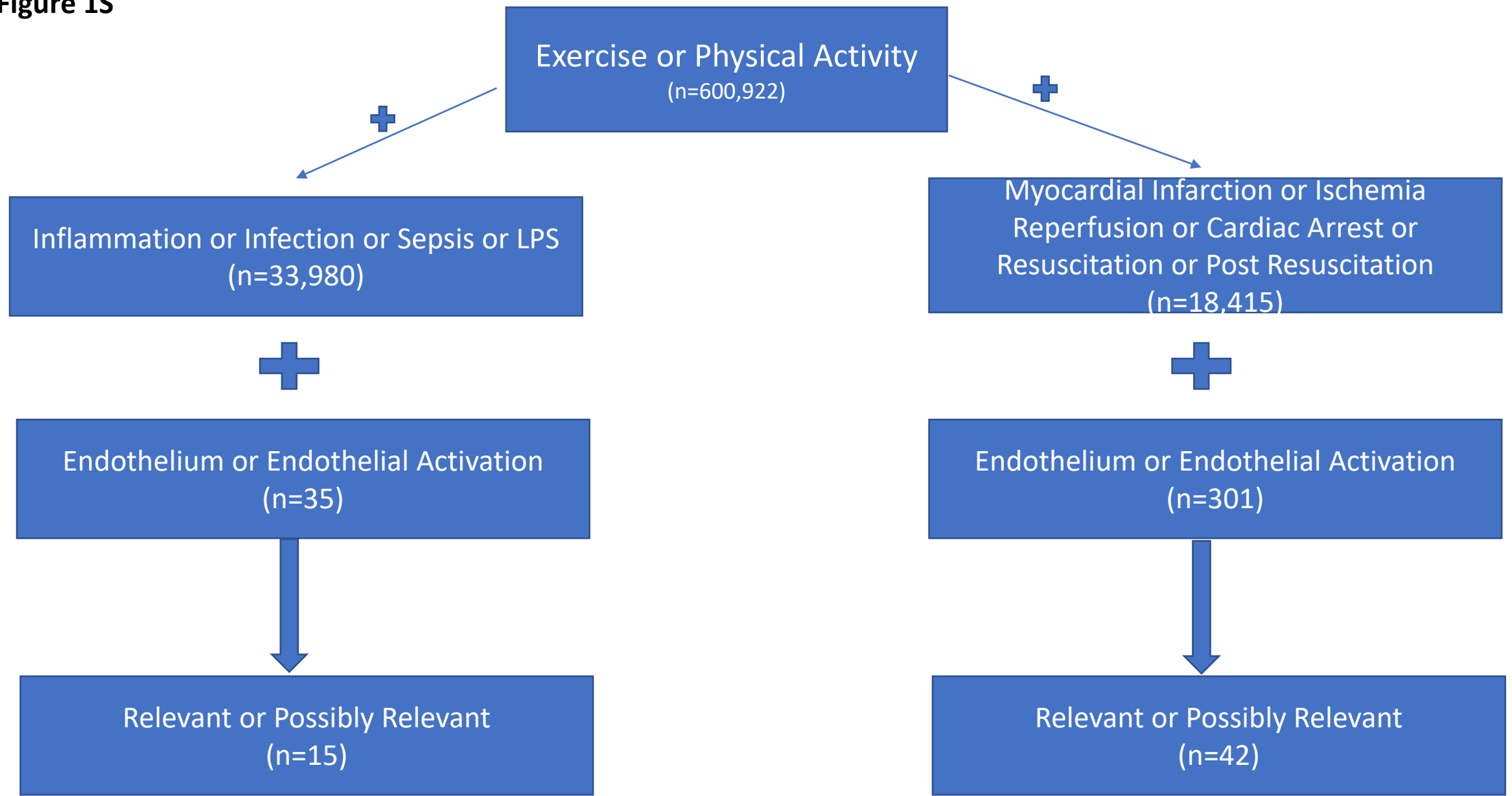

**Figure 2S**

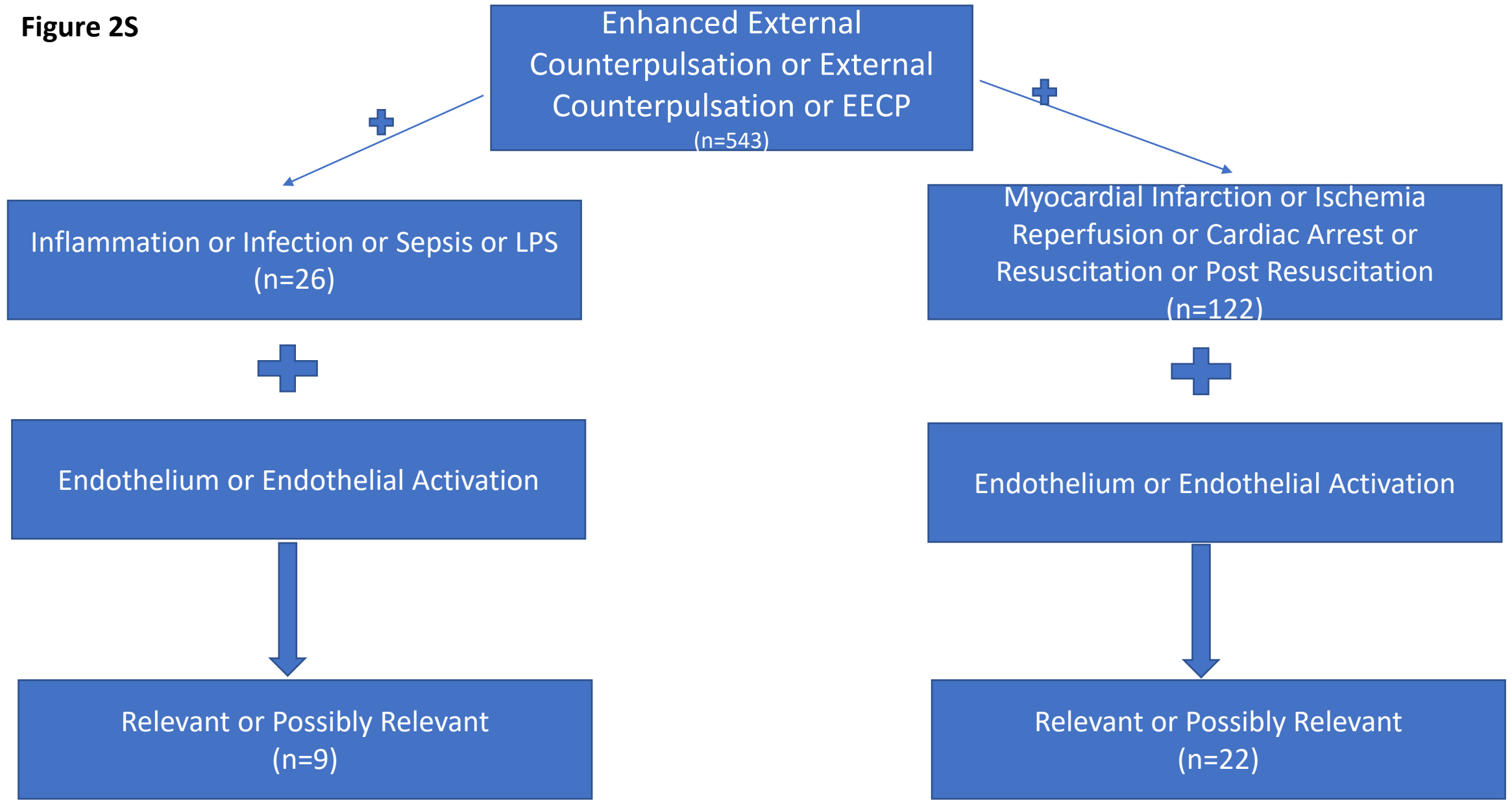

**Figure 3S**

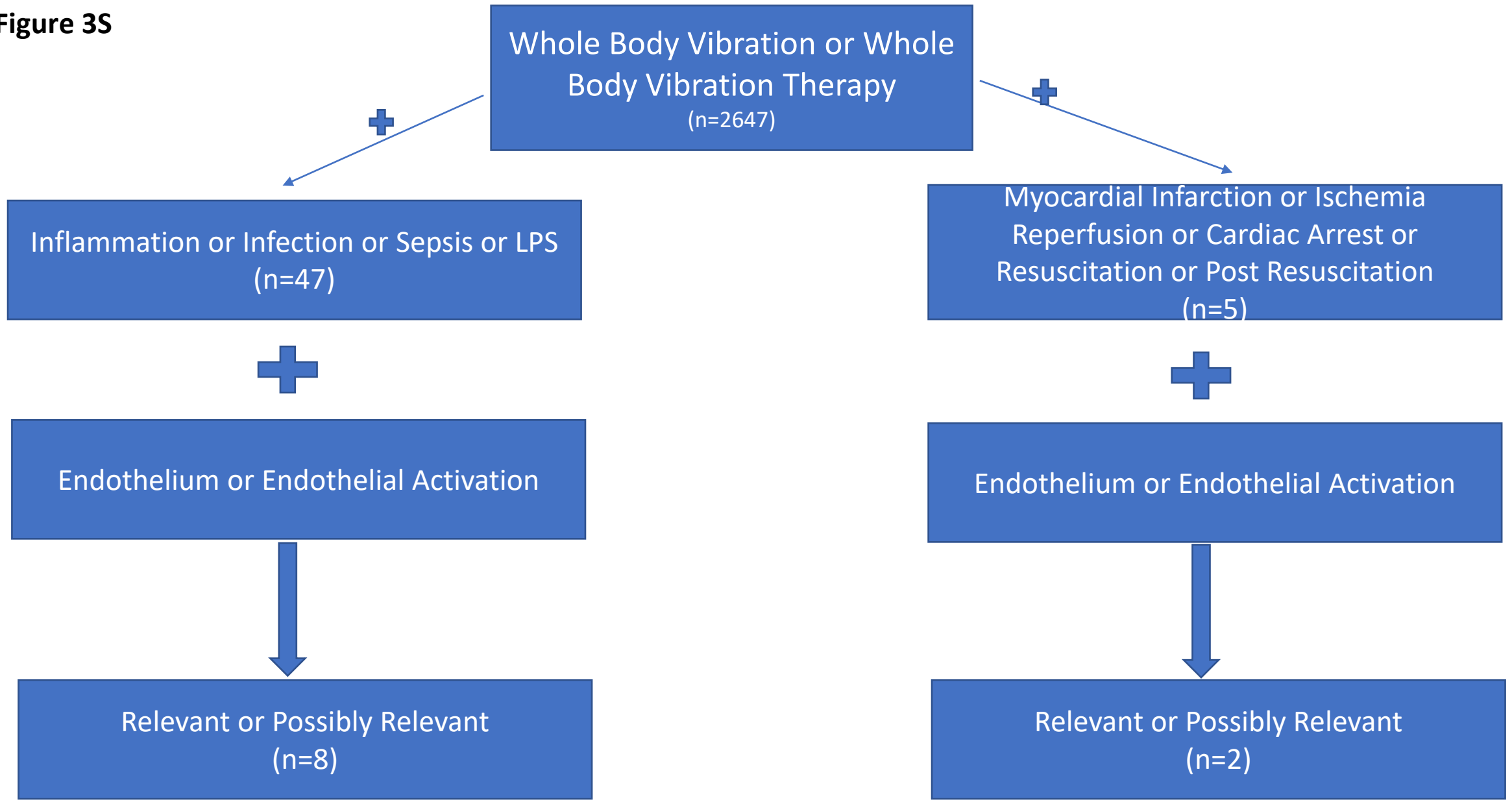

**Figure 4S**

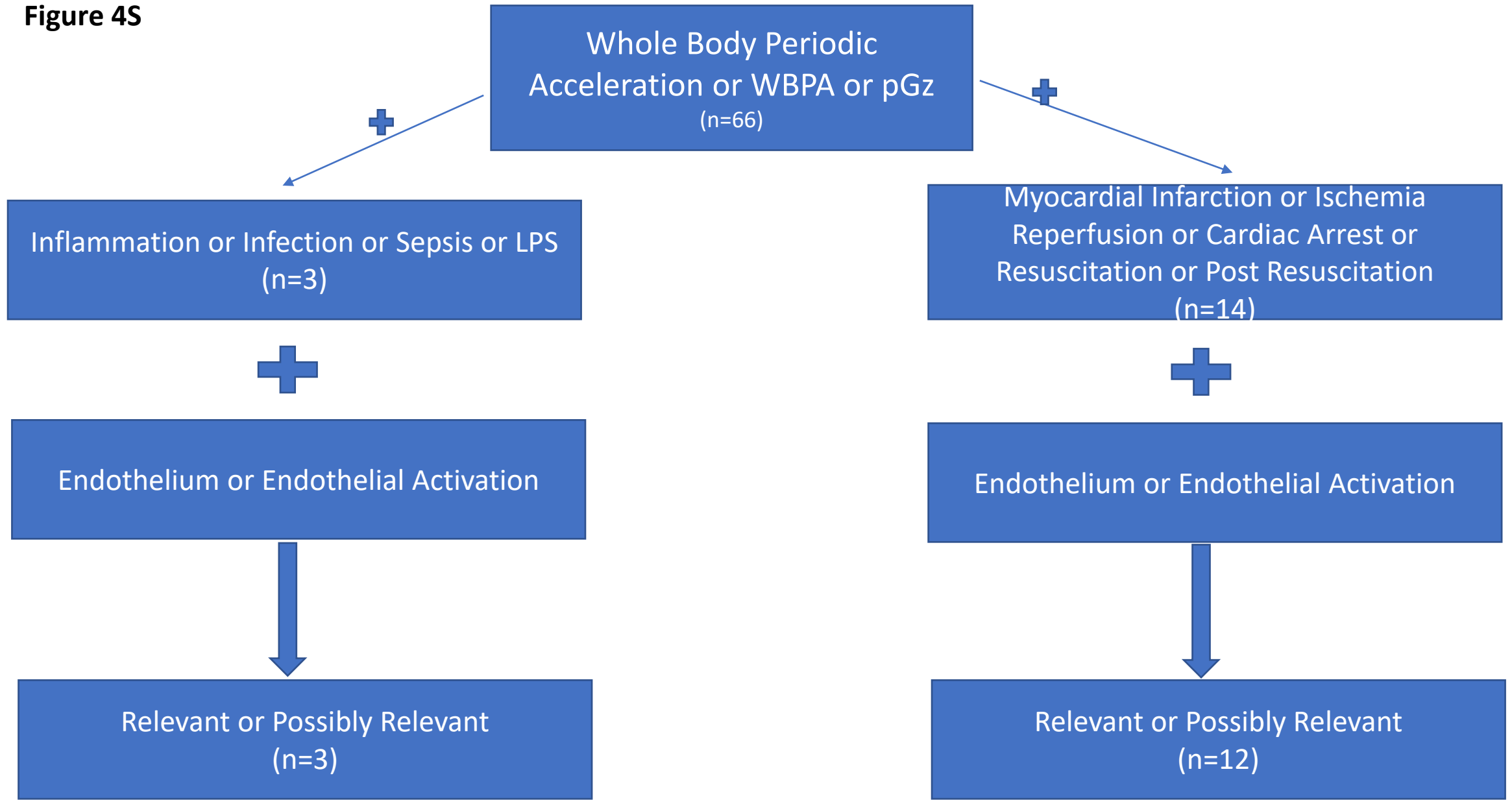

Supplement: Supplementary file 1 [file biomedicines-10-03050-s001.zip › biomedicines-2006007-supplementary.pdf]
